# Maternal urinary manganese and risk of low birth weight: a case–control study

**DOI:** 10.1186/s12889-016-2816-4

**Published:** 2016-02-12

**Authors:** Wei Xia, Yanqiu Zhou, Tongzhang Zheng, Bin Zhang, Bryan A. Bassig, Yuanyuan Li, John Pierce Wise, Aifen Zhou, Yanjian Wan, Youjie Wang, Chao Xiong, Jinzhu Zhao, Zhengkuan Li, Yuanxiang Yao, Jie Hu, Xinyun Pan, Shunqing Xu

**Affiliations:** Key Laboratory of Environment and Health (HUST), Ministry of Education & Ministry of Environmental Protection, and State Key Laboratory of Environmental Health (Incubation), school of Public Health, Tongji Medical College, Huazhong University of Science and Technology, Wuhan, Hubei People’s Republic of China; Department of Environmental Health Sciences, Brown School of Public Health, Providence, RI USA; Women and Children Medical and Healthcare Center of Wuhan, Wuhan, Hubei People’s Republic of China; Department of Environmental Health Sciences, Yale School of Public Health, New Haven, CT USA; Wise Laboratory of Environmental and Genetic Toxicology, Portland, ME USA; Macheng Maternal and Child Health Care Hospital, Macheng, Hubei People’s Republic of China; Ezhou Maternal and Child Health Hospital, Ezhou, Hubei People’s Republic of China

**Keywords:** Manganese, Low birth weight, Maternal urine, Fetal

## Abstract

**Background:**

Manganese (Mn) is an essential element for humans, but exposure to high levels has been associated with adverse developmental outcomes. Early epidemiological studies evaluating the effect of Mn on fetal growth are inconsistent.

**Methods:**

We investigated the association between maternal urinary Mn during pregnancy and the risk of low birth weight (LBW). Mn concentrations in maternal urine samples collected before delivery were measured in 816 subjects (204 LBW cases and 612 matched controls) recruited between 2012 and 2014 in Hubei Province, China.

**Results:**

The median Mn concentration in maternal urine was 0.69 μg/g creatinine. Compared to the medium tertile of Mn levels, an increased risk of LBW was observed for the lowest tertile (≤0.30 μg/g creatinine) [adjusted odds ratio (OR) = 1.28; 95 % confidence interval (CI) = 0.67, 2.45], and a significantly increased risk of LBW was observed for the highest tertile (≥1.16 μg/g creatinine) [adjusted OR = 2.04; 95 % CI = 1.12, 3.72]. A curvilinear relationship between maternal urinary Mn and risk of LBW was observed, showing that the concentration at 0.43 μg/g creatinine was the point of inflection. Similar associations were observed among the mothers with female infants and among the younger mothers < 28 years old. However, among the mothers with male infants or the older mothers ≥ 28 years old, only higher levels of Mn were positively associated with LBW.

**Conclusions:**

Lower or higher levels of maternal urinary Mn are associated with LBW, though only the association of LBW risk and higher levels of Mn was statistically significant. The findings also show that the associations may vary by maternal age and infant sex, but require confirmation in other populations.

**Electronic supplementary material:**

The online version of this article (doi:10.1186/s12889-016-2816-4) contains supplementary material, which is available to authorized users.

## Background

Low birth weight (LBW), a birth weight less than 2,500 g, constitutes a significant public health problem in both developing and developed countries. The estimated 30 million LBW infants born annually (23.8 % of all births) worldwide often face severe short- and long-term health consequences [1]. LBW is not only a major determinant of mortality, morbidity and disability in infancy and childhood [2], but is also associated with a number of health problems in later life, such as neurodevelopmental disabilities, growth and metabolic disorders, and respiratory disorders [3]. There are growing concerns about the role of environmental exposures in the etiology of LBW.

Manganese (Mn) poses a particular challenge in that it is an essential nutrient for normal development, with involvement in bone formation, protein and energy metabolism, and metabolic regulation, but it is also a potential toxicant if overexposure occurs [4]. Mn occurs naturally in the environment and has several industrial uses including production of steels, portable batteries, and aluminum beverage cans. The general population is exposed to Mn through consumption of food and water, inhalation of air, and contact with consumer products that contain Mn. Human activities such as mining, metal smelting, and other industrial uses of Mn may increase Mn levels in the environment [5]. An increasing number of studies suggest that excess exposure to Mn may have detrimental effects on the developing organism, particularly neurological development [6, 7]. Also, maternal exposure to high levels of Mn through drinking water in Bangladesh has been related to increased fetal abnormalities and fetal mortality [8].

Unlike other toxic metals like lead, which have no beneficial use in the human body, the effects of Mn on fetal growth are likely to be more complex because it is both an essential nutrient and a potential toxicant, depending on the amount of exposure. Findings from studies evaluating prenatal exposure to Mn and birth size have been inconsistent. Maternal exposure to elevated levels of Mn in water has been associated with a reduction in birth weight [9]. However, some studies evaluating birth weight and maternal blood Mn levels have observed no such association [10, 11], and a previous study indicated that lower maternal blood Mn levels were associated with intrauterine growth retardation [12]. A recent study evaluated the association in 470 mother-infant pairs and found that both lower and higher maternal blood Mn concentrations were associated with decreased birth weight [13]. Some other studies subsequently reported similar results and suggested that there may be a parabolic dose–response relationship between maternal blood Mn concentrations and birth weight [14–16].

These studies on Mn exposure and infant birth weight were limited to only include normal birth weight infants or included only a few LBW infants in their study populations and therefore couldn’t estimate the risk for LBW. With the high rate of industrial development and urban expansion in China, elevated levels of Mn in air and soil have been reported in some areas [17, 18], which necessitates further study of the potential effects of this element on the developing fetus. Given this background, we conducted a nested case–control study that included 204 LBW cases and 612 matched controls in Hubei Province, China. Assessment of Mn exposure levels in pregnant women can be as a surrogate of exposure to the unborn child, because Mn can cross the placenta and transfer from the mother to the fetus [19]. Noninvasive urine based analysis is a traditional approach for evaluating trace-elements levels in the human body. Several studies conducted worldwide used Mn in urine to indicate occupational or environmental exposure for assessing health risks [20–22], and found associations between urinary Mn levels and adverse health outcomes [20, 23, 24]. In the present study, we examined the association between maternal urinary Mn concentrations and the risk of LBW. Potential effect modification by maternal age and infant sex, which has not been previously evaluated, was also examined.

## Methods

### Study population

The subjects in this study were participants in the prospective Healthy Baby Cohort (HBC) study. This study was conducted at three major maternity hospitals in Wuhan, Ezhou, and Macheng cities, which are located in Hubei province, in the central of People’s Republic of China. Between November 2012 and April 2014, 16,293 women who gave birth at any of the three hospitals were recruited, and the participation rate was 78.7 %. Participant mothers received a detailed explanation of the study procedures and provided written informed consent at enrollment. The research protocol was approved by the ethical committee of Tongji Medical College, Huazhong University of Science and Technology, and the three study hospitals.

In this study, cases were mothers who delivered a singleton live infant with a birth weight < 2,500 g. Controls were mothers who delivered a singleton live infant with normal birth weight between ≥ 2,500 g and < 4,000 g. Potential cases and controls were excluded if they gave birth to multiple infants, a stillborn infant, or an infant with a birth defect. Women who did not have urine samples available for analysis were also excluded. For each case selected, three consecutive individual controls were randomly selected and matched to one case by delivery hospital, infant sex, and maternal age at conception (within 1-year interval). If more suitable matched controls were available for one case, only the top three with the closest maternal age to the case were selected. A total of 204 cases and 612 matched controls were included in the analysis.

### Data collection

Women were interviewed after delivery by specially trained nurses in the three hospitals. The questionnaire collected information regarding demographic factors, household income, education, smoking behavior, and alcohol consumption. Information on birth outcomes, reproductive history, disease, and pregnancy complications were abstracted from the medical records. The pre-pregnancy body mass index (BMI) of mothers was calculated using the self-reported weight before pregnancy and height, which was measured using a stadiometer. Gestational age was calculated by subtracting the date for the last menstrual period from the date of delivery. Infant birth weight was measured by delivery room staff using standardized anthropometric procedures.

### Urinary Mn measurements

The maternal urine samples were obtained during the hospital admission for delivery, and were collected in polypropylene tubes and stored at −20 °C prior to analysis. All samples were coded and analyzed by lab personnel blind to their origin. Urine samples were thawed at room temperature before analysis, and 1 mL of urine from the supernatant was introduced in a Kirgen polypropylene conical centrifuge tubes. Then, 3 % HNO_3_ was added to the final volume of 5 mL for overnight nitrification. The resulting sample was digested by ultrasound at 40 °C for 1 h and then analyzed using inductively coupled plasma mass spectrometry (Agilent 7700, Agilent Technologies, Santa Clara, CA, USA). The standard Reference Material Human Urine (SRM2670a, National Institute of Standards and Technology, Gaithersburg, MD, USA) was used as an external quality control, and sample spike-recoveries were used to confirm analytical recovery, which was 95 %. A 3 % HNO_3_ blank was processed in each batch of samples to control for possible contamination. The samples were analyzed with an external calibration method, using eight standard concentrations ranging from 0 to 500 μg/L. The limit of detection (LOD) for Mn in urine was 0.05 μg/L. Field blanks were also included for quality control and the levels of Mn in the field blanks were < LOD. The urine samples below the LOD were given a value one-half the LOD. Lead, arsenic, cadmium, and thallium were also measured simultaneously because previous studies have suggested that these metals are potentially associated with decreasing birth weight [25–28].

Urinary creatinine concentrations were measured using a commercially available diagnostic enzyme method (Mindray BS-200 CREA Kit, Shenzhen Mindray Bio-medical Electronics CO., LTD., Shenzhen, China). Urine creatinine was used to adjust the urinary Mn concentrations (μg/L) in order to correct for urine dilution, and Mn concentrations were expressed as μg/g creatinine.

### Statistical analysis

The Wilcoxon signed rank test was used to compare distributions of Mn levels between cases and controls, because the distribution of maternal urinary Mn was skewed to the right. Conditional logistic regression analyses were performed to assess the association between maternal urinary Mn concentrations and risk of LBW. Maternal urinary Mn levels were analyzed as categorical variables based on the tertile distribution of Mn concentrations in the controls. Crude and adjusted odds ratios (ORs) and their 95 % confidence intervals (CIs) were estimated comparing the lowest and highest tertile of Mn levels to the medium tertile. Household income was used to represent socioeconomic status in this study, because adjustment for income had a larger impact on the estimate than education. Inclusion of the two variables together in the adjusted model did not produce significantly different results compared to the addition of each individual variable into the model separately. In the final model, we adjusted for gestational age (<37 weeks, ≥ 37 weeks), household income (≥50,000, < 50,000 yuan per year), gestational hypertension (No, Yes), pre-pregnancy body mass index (<18.5, 18.5–23.9, ≥ 24), parity (primiparous, multiparous), and passive smoking (No, Yes). Additional adjustment for occupational status, diabetes, and multivitamin supplement use during pregnancy did not result in material changes in the observed associations and thus were not included in the final models. Several metals that have been previously suggested to be associated with birth weight (lead, arsenic, cadmium, and thallium) were also adjusted for in the models to control for potential confounding, but inclusion of these metal variables either individually or together did not cause a significant change in the risk estimates associated with maternal urinary Mn concentrations. Smoking and alcohol consumption during pregnancy were not included because very few Chinese women smoke and drink throughout life. Risk estimates were stratified by infant sex and maternal age, and heterogeneity of effects by infant sex and maternal age were assessed by the Breslow–Day test. In addition, we applied the SAS macro LGTPHCURV8 [29] to fit restricted cubic splines to conditional logistic model to examine the possible non-linear relationship between maternal urinary Mn concentrations and risk of LBW. Maternal urinary Mn was regarded as a continuous variable, and the referent value was set to the median. The output showed the significance level from the likelihood ratio tests for non-linearity or linearity, and the results of spline or linear model were plotted. A two-sided *P* value of < 0.05 was considered statistically significant. All data analyses were performed using SAS (version 9.3; SAS Institute Inc., Cary, NC, USA).

## Results

General characteristics of the cases and controls are presented in Table 1. Of the newborn infants, 49.5 % were male. The mean maternal age at delivery was 28.1 ± 4.7 years with a range of 17 to 42 years. Compared to the controls, the case mothers were more likely to have fewer years of education, have a lower pre-pregnancy body mass index, and report a lower household income. There were higher proportions of case mothers who had gestational hypertension. Only one mother reported smoking and only three mothers reported drinking alcohol during their pregnancy.

**Table 1:** Basic characteristics of low birth weight cases and controls [*n* (%)]

Characteristics	Cases (*n* = 204)	Controls (*n* = 612)
Infant sex		
Male	101 (49.5)	303 (49.5)
Female	103 (50.5)	309 (50.5)
Maternal age		
< 25 years	48 (23.5)	146 (23.8)
25–29 years	81 (39.7)	242 (39.5)
≥ 30 years	75 (36.7)	224 (36.6)
Education		
≤ 9 years	89 (43.6)	167 (27.3)
9–12 years	38 (18.6)	120 (19.6)
> 12 years	77 (37.8)	322 (52.6)
Missing	0 (0.0)	3 (0.5)
Household income		
< 50,000 yuan per year	116 (56.9)	275 (44.9)
≥ 50,000 yuan per year	61 (29.9)	279 (45.6)
Missing	27 (13.2)	58 (9.5)
Parity		
Primiparous	159 (77.9)	501 (81.9)
Multiparous	45 (22.1)	111 (18.1)
Gestational hypertension		
Yes	20 (9.8)	12 (2.0)
No	183 (89.7)	598 (97.7)
Missing	1 (0.5)	2 (0.3)
Gestational diabetes		
Yes	7 (3.4)	27 (4.4)
No	197 (96.6)	585 (95.6)
Pre-pregnancy BMI		
< 18.5 kg/m^2^	60 (29.4)	125 (20.4)
18.5–23.9 kg/m^2^	108 (52.9)	385 (62.9)
≥ 24 kg/m^2^	26 (12.8)	85 (13.9)
Missing	10 (4.9)	17 (2.8)
Multivitamin supplement during pregnancy		
Yes	137 (67.2)	409 (66.8)
No	59 (28.9)	190 (31.1)
Missing	8 (3.9)	13 (2.1)
Smoking during pregnancy		
No	198 (97.1)	608 (99.4)
Yes	1 (0.5)	0 (0.0)
Missing	5 (2.4)	4 (0.6)
Passive smoking during pregnancy		
Yes	47 (23.0)	129 (21.1)
No	149 (73.0)	464 (75.8)
Missing	8 (3.9)	19 (3.1)
Alcohol use during pregnancy		
Yes	2 (1.0)	1 (3.3)
No	196 (96.1)	594 (97.1)
Missing	6 (2.9)	17 (2.8)

The median Mn concentration in maternal urine was 0.69 μg/g creatinine (Additional file 1: Table S1). The LBW case mothers had significantly higher urinary Mn levels compared to the control mothers (median: 1.09 vs. 0.64 μg/g creatinine, *P* < 0.01). Older mothers ≥ 28 years old had elevated Mn concentrations compared to younger mothers < 28 years old among the controls (median: 0.67 vs. 0.59 μg/g creatinine) and the cases (median: 1.14 vs. 1.01 μg/g creatinine), although these differences were not statistically significant (*P* > 0.05). Further, Mn concentrations were somewhat higher in the mothers that gave birth to male infants compared to female infants among the controls (median: 0.68 vs. 0.59 μg/g creatinine, *P* > 0.05) and the cases (median: 1.19 vs. 0.93 μg/g creatinine, *P* > 0.05).

**Fig. 1:**
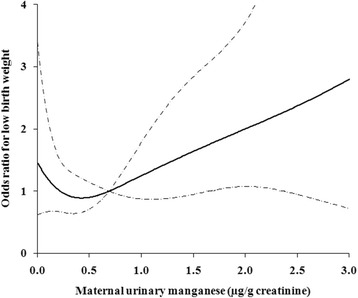
The relationship between maternal urinary manganese concentration (μg/g creatinine) and risk of LBW, adjusted for gestational age, household income, pre-pregnancy body mass index, parity, passive smoking, and gestational hypertension. The risk estimate is indicated by the solid line, and the 95 % confidence intervals are represented by the dashed lines

Table 2 shows the association between maternal urinary Mn levels and LBW risk. Compared to the medium tertile of Mn concentration (0.30-1.16 μg/g creatinine), both the lowest and highest tertiles of Mn were associated with increased risk of LBW in the adjusted analysis, although the association was only significant for the highest tertile [adjusted OR = 1.28 (95 % CI: 0.67, 2.45) for the lowest tertile; adjusted OR = 2.04 (95 % CI: 1.12, 3.72) for the highest tertile]. In the restricted spline model after adjusting for the potential confounders, we observed a U-shaped relationship with a concentration at 0.43 μg/g creatinine as the point of inflection between maternal urinary Mn and risk of LBW (Fig. 1). However, the non-linear relationship was not significant (*P* = 0.12).

**Table 2:** Risk of low birth weight associated with levels of manganese in maternal urine

Manganese (μg/g creatinine)	Cases	Controls	OR^a^ (95 % CI)	OR^b^ (95 % CI)
Total (*n* = 816)				
< 0.30	55	204	1.11 (0.71, 1.74)	1.28 (0.67, 2.45)
0.30–1.16	48	204	1.00	1.00
≥ 1.16	101	204	2.19 (1.46, 3.27)	2.04 (1.12, 3.72)

Abbreviation: *OR* odds ratio; *CI* confidential interval

^a^ Unadjusted odds ratio

^b^ Adjusted for gestational age, household income, pre-pregnancy body mass index, parity, passive smoking, gestational hypertension

Results for stratified analyses by maternal age (<28 and ≥ 28 years) and infant sex are shown in Table 3 and Table 4, respectively. Among younger mothers (<28 years), both lower and higher Mn concentrations in urine were associated with increased risk of delivering LBW infants, and the association was significant for the highest tertile [adjusted OR = 1.88 (95 % CI = 0.74, 4.80) for the lowest tertile; adjusted OR = 2.74 (95 % CI = 1.07, 7.03) for the highest tertile]. Although no significant interaction between maternal age and maternal urinary Mn levels was observed (*P* heterogeneity = 0.19), a positive association with LBW was only observed for the highest tertile among older mothers (≥28 years) [adjusted OR = 1.98 (95 % CI = 0.82, 4.74)]. For mothers that gave birth to female infants, lower and higher Mn levels were associated with increased risk of LBW compared to the medium tertile [adjusted OR = 2.12 (95 % CI: 0.86, 5.25) for the lowest tertile; adjusted OR = 1.94 (95 % CI: 0.88, 4.45) for the highest tertile], although the associations were not significant. For mothers that gave birth to male infants, a positive association with LBW was only observed for the highest tertile of Mn [adjusted OR = 1.88 (95 % CI: 0.85, 4.79)]. Also, there was no statistical evidence of heterogeneity in risk according to infant sex (*P* heterogeneity = 0.24). Moreover, we obtained consistent results with the restricted spline models, showing that a non-linear risk pattern in younger mothers and mothers who gave birth to female infants (Additional file 1: Figure S1), though the non-linear relationship was only significant in mothers who gave birth to female infants (*P* = 0.82 and *P* = 0.02, respectively). For older mothers and mothers who gave birth to male infants, we observed a possible linear trend between maternal urinary Mn concentrations and risk of LBW (*P* = 0.03 and *P* = 0.08, respectively).

**Table 3:** Risk of low birth weight associated with maternal urinary manganese levels, stratified by maternal age

Manganese levels^a^	Age < 28 years old (*n* = 392)			Age ≥ 28 years old (*n* = 424)			*P* for heterogeneity
	Ca/Co	OR^b^ (95 % CI)	OR^c^ (95 % CI)	Ca/Co	OR^b^ (95 % CI)	OR^c^ (95 % CI)	
Tertile 1	31/98	1.80 (0.91, 3.56)	1.88 (0.74, 4.80)	23/106	0.72 (0.39, 1.34)	0.84 (0.31, 2.24)	0.19
Tertile 2	17/98	1.00	1.00	31/106	1.00	1.00	
Tertile 3	50/98	3.14 (1.67, 5.92)	2.74 (1.07, 7.03)	52/106	1.69 (0.98, 2.90)	1.98 (0.82, 4.74)	

Abbreviations: ca/co, numbers of cases and controls; *CI* confidence interval; *OR* odds ratio

^a^ Manganese levels (μg/g creatinine): age < 28 years, tertile 1 (<0.30), tertile 2 (0.30–1.08), tertile 3 (≥1.09); age ≥ 28 years, tertile 1 (<0.28), tertile 2 (0.28–1.21), tertile 3(≥1.22)

^b^ Crude odds ratio

^c^ Adjusted for gestational age, household income, pre-pregnancy body mass index, parity, passive smoking, and gestational hypertension

**Table 4:** Risk of low birth weight associated with maternal urinary manganese levels, stratified by infant sex

Manganese levels^a^	Male (*n* = 404)			Female (*n* = 412)			*P* for heterogeneity
	Ca/Co	OR^b^ (95 % CI)	OR^c^ (95 % CI)	Ca/Co	OR^b^ (95 % CI)	OR^c^ (95 % CI)	
Tertile 1	22/101	0.73 (0.38, 1.40)	0.64 (0.22, 1.83)	32/103	1.55 (0.81, 2.98)	2.12 (0.86, 5.25)	0.24
Tertile 2	29/101	1.00	1.00	20/103	1.00	1.00	
Tertile 3	50/101	1.77 (1.02, 3.06)	1.88 (0.85, 4.79)	51/103	2.65 (1.46, 4.80)	1.94 (0.88, 4.45)	

Abbreviations: ca/co, numbers of cases and controls; *CI* confidence interval; *OR* odds ratio

^a^ Manganese levels (μg/g creatinine): male, tertile1 (<0.31), tertile 2 (0.31–1.24), tertile 3 (≥1.25); female, tertile 1 (<0.28), tertile 2 (0.28–1.04), tertile 3(≥1.05)

^b^ Crude odds ratio

^c^ Adjusted for gestational age, household income, pre-pregnancy body mass index, parity, passive smoking, and gestational hypertension

## Discussion

In this nested case–control study, we observed an increased risk of LBW for maternal urinary Mn levels in the lowest tertile and highest tertile, though the associations with LBW risk were only significant for higher levels of urinary Mn. These associations were observed even after adjustment for known and suspected risk factors for LBW. We further identified a curvilinear relationship between maternal urinary Mn level and risk of LBW, which was apparent in female infants. The observed relationship was consistent with our *a priori* hypothesis, and supports the idea that Mn may contribute to adverse birth outcomes at both low and high exposure levels.

The number of studies on biomarkers of Mn has been increased during recent two decades, but the results on the representative biomarkers of Mn are contradictory, including Mn in urine, blood and hair. Some studies showed that urinary Mn may have limited use for occupational population as a direct measure of Mn exposure due to high variability within person over time [30, 31]. However, previous studies also have shown that Mn detected in spot urine sample is able to correctly classify individuals into the higher levels of Mn vs. the lower levels of Mn exposure [20, 21]. Since behavior, consumption habits, and ambient living environment of individuals’ do not change easily, we used urinary Mn concentration to divide the study populations into three levels of exposure to investigate its association with risk of LBW. Also, elevated urinary concentrations have been reported to correlate to elevated concentrations of Mn in drinking water in pregnant women living in Bangladeshi, suggesting maternal urine may serve as a useful Mn biomarker to estimate prenatal exposure [22].

In the present study, none of our study participants had a urinary Mn level higher than the upper limit of normal urinary Mn values for adults (10 μg/L) [32]. Table 5 shows the comparison of Mn concentrations in urine from this study and previous studies in pregnant women and general population around the world. The results showed that the Mn concentrations in maternal urine observed in this study (median 0.38 μg/L, 0.69 μg/g creatinine) were comparable to those seen in pregnant women in western Australia (median 0.33 μg/L, 0.53 μg/g creatinine) [33] and California, USA (0.40 μg/L) [34]. Compared to the pregnant women living in rural Bangladesh with higher Mn concentrations in drinking water than other places (median 1.60 μg/L) [22], our study population had much lower urinary Mn concentrations. Previous studies have shown maternal blood Mn levels were increased during pregnancy [10, 35, 36]. The increase in Mn levels during pregnancy may be related to physiological factors, including increased intestinal absorption or tissue Mn mobilization [37, 38], reflecting increased need from the fetal development. Takser et al. [10] reported that blood Mn concentration in pregnant women at delivery was higher than non-pregnant women of reproductive age. In consistent with the findings, Mn concentrations in urine of pregnant women in this study were a little higher than those observed in the healthy women from Canada (median 0.09 μg/L) [39], Japan (median 0.16 μg/L, 0.13 μg/g creatinine) [40], as well as the general population (including men and women) in France (median 0.31 μg/L) [41] and Germany (arithmetic mean 0.09 μg/L) [42].

**Table 5:** Comparison of manganese concentrations in urine from the present study and previous studies

						Percentiles	
Location	Author (year)	Population	Number	Arithmetic Mean	25th	50th	75th
Hubei, China	Present study (2014)	Pregnant women	816	0.66 μg/L 1.12 μg/g creatinine	0.13 μg/L 0.18 μg/g creatinine	0.38 μg/L 0.69 μg/g creatinine	0.85 μg/L 1.87 μg/g creatinine
Western Australia	Callan (2013) [33]	Pregnant women	173	1.00 μg/L 1.22 μg/g creatinine	——	0.33 μg/L 0.53 μg/g creatinine	——
California, USA	Gunier (2014) [34]	Pregnant women	59	——	0.20 μg/L	0.40 μg/L	0.60 μg/L
Bangladeshi	Ljung (2009) [22]	Pregnant women	388	2.50 μg/L	0.90 μg/L	1.60 μg/L	2.80 μg/L
Canada	Health Canada (2010) [39]	Women	5309	0.15 μg/L	< LOD	0.09 μg/L	0.17 μg/L
Japan	Ohashi (2006) [40]	Women	1000	——	——	0.16 μg/L 0.13 μg/g creatinine	——
France	Goullé (2005) [41]	General population	100	——	——	0.31 μg/L	——
Germany	Heitland (2006) [42]	General population	87	0. 09 μg/L	——	——	——

—— No data

Currently, there are no recommended values or guidelines for ideal urinary or blood Mn levels during pregnancy. In this study, we used the medium tertile of urinary Mn as the reference group, and observed that both the lowest and highest tertile were positively associated with LBW. One explanation for the weaker association between the lowest tertile of Mn and LBW risk might be that the prevalence of Mn deficiency is low in our population. In a previous study, Eum et al. [15] assessed the relationship between maternal blood Mn levels before delivery and birth weight categorized as a binary variable (below 3000 g or more than 3000 g) in 331 full-term infants. They used the medium quintile as the reference group and found that the lowest (<16.9 μg/L) and the highest quintiles (≥26.9 μg/L) of blood Mn were associated with an increased risk of delivering infants with a birth weight below 3000 g. However, the associations were not significant for the lowest quintile (adjusted OR = 2.77; 95 % CI: 0.89, 8.65) and the highest quintile of Mn levels (adjusted OR = 2.66; 95 % CI: 0.84, 8.08). Given that the risks observed in that study were in relation to birth weights < 3000 g, it is possible that they didn’t include LBW infants or only included a small number of LBW infants in the study.

Some previous studies also investigated the relationship between maternal Mn exposure and infant birth weight. Vigeh et al. [13] reported that intrauterine growth retardation was positively associated with maternal blood Mn and negatively associated with cord blood Mn in 271 mother-infant pairs from Tehran, Iran, but the study did not evaluate non-linear relationships. Zota et al. [13] found a non-linear relationship between maternal Mn blood Mn levles and birth weight in a cohort of 470 mother–infant pairs from Oklahoma. They reported an inverted U-shaped relationship between maternal blood Mn levels and birth weight with a concentration of 31 μg/L as the point of inflection. The report of Eum et al. [15] found a consistent result that the infant birth weight increased with maternal blood Mn concentration up to 35 μg/L, and then decreased. Chen et al. [14] also obtained similar results in a study of 172 mother-infant pairs in Shanghai in that an inverted U-shaped relationship was found between maternal blood Mn levels and birth weight. A similar parabolic dose–response relationship between cord blood Mn concentrations and birth weight was observed by Guan et al. [16] in Dalian City, northern China (*n* = 125). Interestingly, in consistent with the studies, we also found a potential U-shaped relationship between maternal urinary Mn level and risk of LBW. However, the non-linear relationship may vary by maternal age and infant sex, and the non-linear pattern was apparent in mothers who gave birth to female infants.

Infant sex is known to influence pregnancy outcomes, and female infants are usually at higher risk of LBW since male infants have consistently higher birth weight throughout gestation compared to female infants [43]. However, the relationship between maternal Mn levels and infant sex has not been studied before. We observed that the mothers who gave birth to male infants had slightly higher Mn concentrations compared to mothers who gave birth to female infants, which is one possible explanation for why only higher but not lower Mn levels were associated with LBW risk among male infants. In addition, maternal age has been regarded as a factor that can affect blood Mn levels [18, 44], and a previous study also reported that urinary Mn levels increased at advanced ages [40]. In our analyses stratified by maternal age, only higher Mn exposure levels were associated with LBW risk among older mothers, whereas for younger mothers both lower and higher Mn levels were associated with an elevated LBW risk. A reason for this difference might be that the older mothers had elevated Mn concentrations compared to younger mothers, though there was no significant difference. The potential differences in the effect of Mn exposure on LBW according to sex and maternal age observed in this study should be interpreted carefully due to the insufficient sample size, and further studies are needed to confirm the results.

With respect to maternal exposure to higher levels of Mn being associated with LBW, one biologically plausible mechanism could be oxidative stress caused by high levels of Mn, leading to impairment of cellular function and growth [45]. Experimental studies on fetal development also showed that maternal exposure to high levels of Mn through oral administration resulted in a decrease in fetal weight and retardation of the development of the skeleton and internal organs [46]. There is also a concern that Mn deficiency may cause adverse effects on fetal growth, in that it is a critical component of the bone matrix and an important cofactor for enzymes necessary for bone metabolism [47]. In animals, the main features of Mn deficiency are skeletal malformation and impaired growth [48, 49]. Dietary deficiency of Mn has been associated with abnormal glucose tolerance and perturbation of carbohydrate metabolism in animal studies [50], which could also contribute to fetal growth restriction. More research is needed to assess the effect of Mn exposure during pregnancy on subsequent child development.

One of the strengths of our study is that the nested case–control design provided the opportunity to include all of the LBW infants in the cohort, and the cases and controls were matched on potentially important factors in order to reduce the influence of confounding. The other strength of our study includes the availability of interview data and medical records from all participants, which allowed us to adjust for other potential risk factors for LBW.

The limitation of this study is that Mn levels were measured in maternal urine at one spot time, which may not accurately reflect maternal Mn load or fetal exposure during the entire pregnancy. Biliary excretion is the main pathway by which Mn is excreted with most of the element ultimately being excreted in the feces [51], and urinary Mn excretion representing about 5 % of the total excreted amount [52]. Recent studies suggested that levels of Mn measured in tooth dentin, toenails or cord blood may constitute valid biomarkers of early life exposure [34, 53]. Future studies are needed that use these biomarkers to investigate Mn exposure and birth outcomes. In addition, repeated measures of erythrocytes Mn over trimesters may also provide an effective biomarker for Mn exposure during pregnancy [36]. A prospective study of Mn concentrations measured at different time points during pregnancy may help to evaluate how Mn levels change over the course of the pregnancy and whether there is a critical exposure window for the effect of Mn on fetal development.

## Conclusions

Our nested case–control study found that both lower and higher levels of Mn concentration, as measured in maternal urine before delivery, was associated with increased risk of infant LBW. The associations were further observed to vary by maternal age and infant sex. This observation suggests that Mn levels during pregnancy may be important for fetal growth.

## Additional file

Additional file 1:
**Distribution of maternal urianry manganese and restricted spline curves association between urinary manganese concentration and risk of low birth weigh in the stratified analysis.** (DOCX 104 kb)

## Abbreviations

LBW: Low birth weight
Mn: Manganese
CI: Confidence interval
OR: Odds ratio
BMI: Body mass index
LOD: Limit of detection
