# Supplementary material for: Maternal urinary manganese and risk of low birth weight: a case–control study
Source: BMC Public Health. 2016 Feb 12;16:142. doi: 10.1186/s12889-016-2816-4 (PMC4751650; doi:10.1186/s12889-016-2816-4)
Supplement: Additional file 1: — Distribution of maternal urianry manganese and restricted spline curves association between urinary manganese concentration and risk of low birth weigh in the stratified analysis. (DOCX 104 kb) [file 12889_2016_2816_MOESM1_ESM.docx]

**Supplementary Material**

**Table S1.** The distribution of manganese concentrations in maternal urine (µg/g creatinine).

|  | | Mean | Selected percentiles | | | | |
| --- | --- | --- | --- | --- | --- | --- | --- |
|  |  |  | 5th | 25th | Median | 75th | 95th |
| Total | | 2.12 | <LOD | 0.18 | 0.69 | 2.01 | 8.92 |
|  | Cases | 2.52 | <LOD | 0.24 | 1.09 | 2.94 | 9.16 |
|  | Controls | 1.99 | <LOD | 0.17 | 0.64 | 1.77 | 8.42 |
| Male infants | | 2.27 | <LOD | 0.20 | 0.79 | 2.14 | 9.42 |
|  | Cases | 2.59 | 0.02 | 0.34 | 1.19 | 3.10 | 9.29 |
|  | Controls | 2.17 | <LOD | 0.16 | 0.68 | 1.83 | 9.42 |
| Female infants | | 1.97 | <LOD | 0.17 | 0.65 | 1.98 | 8.51 |
|  | Cases | 2.45 | <LOD | 0.14 | 0.93 | 2.86 | 9.14 |
|  | Controls | 1.80 | <LOD | 0.18 | 0.59 | 1.59 | 7.13 |
| < 28 years old | | 1.99 | <LOD | 0.17 | 0.62 | 1.95 | 8.59 |
|  | Cases | 2.44 | <LOD | 0.16 | 1.01 | 2.77 | 8.69 |
|  | Controls | 1.83 | <LOD | 0.17 | 0.59 | 1.71 | 7.15 |
| ≥ 28 years old | | 2.24 | <LOD | 0.21 | 0.73 | 2.18 | 9.42 |
|  | Cases | 2.59 | 0.01 | 0.34 | 1.14 | 3.32 | 9.29 |
|  | Controls | 2.13 | <LOD | 0.16 | 0.67 | 1.81 | 9.79 |

**Figure S1. The relationship between maternal urinary manganese concentration and risk of LBW in the stratified analysis.** Restricted spline curve association between urinary manganese concentration (μg/g creatinine) and odds of low birth weight in mothers <28 years old (A), mothers ≥ 28 years (B), mothers who gave birth to female infants (C), and mothers who gave birth to female infants (D). All the models were adjusted for gestational age, household income, pre-pregnancy body mass index, parity, passive smoking, and gestational hypertension. The risk estimate is indicated by the solid line, and the 95% confidence intervals are represented by the dashed lines.

**
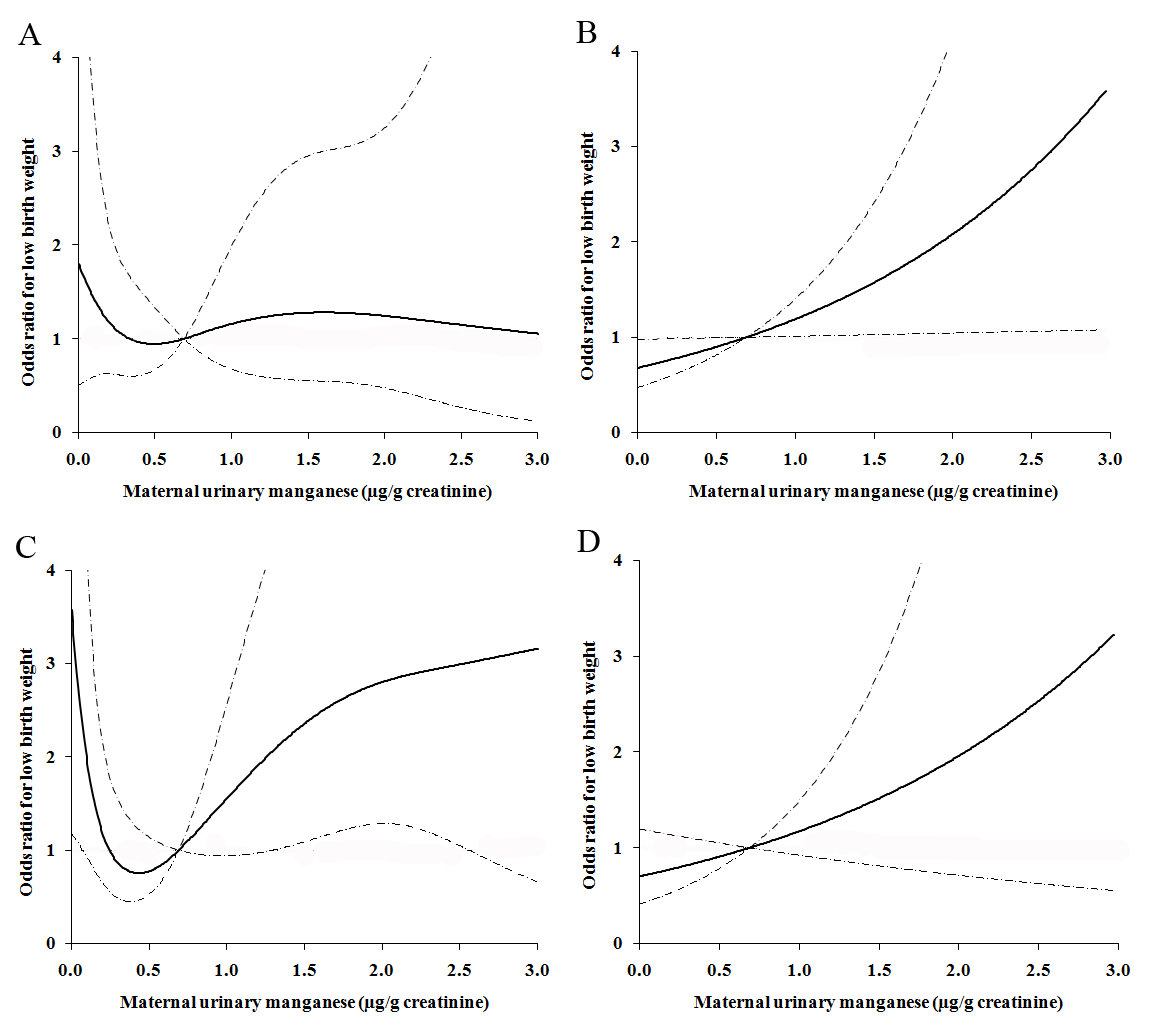
**
